# Lessons Learned on Recruiting and Retaining Young Fathers in a Parenting and Repeat Pregnancy Prevention Program

**DOI:** 10.1007/s10995-020-02956-w

**Published:** 2020-06-20

**Authors:** Sara McGirr, Jennifer Torres, Julia Heany, Hillary Brandon, Carrie Tarry, Christopher Robinson

**Affiliations:** 1 Michigan Public Health Institute, 2342 Woodlake Drive, Okemos, MI 48864 USA; 2 Michigan Department of Health and Human Services, 109 W Michigan Ave, Lansing, MI 48933 USA

**Keywords:** Fatherhood, Young men, Recruitment, Retention, Teenage pregnancy

## Abstract

**Introduction:**

Research shows that mainstream parenting and repeat pregnancy prevention programs generally do not effectively engage with fathers and that young men’s levels of participation in such services are low. To support practitioners in overcoming the barriers to recruiting and retaining young fathers, the current study aimed to gather lessons learned from one program’s state administrators, case managers, and young fathers about the most effective strategies for engaging this population in intensive case management.

**Methods:**

Three focus groups were conducted. One focus group was held with the creators and managers of the Michigan Adolescent Pregnancy and Parenting Program MI-APPP at the state Department of Health and Human Services (n = 3). The other two groups were designed to jointly engage young fathers currently involved in intensive case management (n = 11) and their case managers (n = 5). A qualitative analysis of the focus group transcripts was conducted using a coding scheme developed from emerging themes in the transcripts and related literature.

**Results:**

The findings highlight a selection of those strategies that focus group participants perceived to be most successful in improving male recruitment and retention in intensive ongoing case management. Among these strategies were centralizing feedback from young fathers in program decision making, offering opportunities for young fathers to connect, and challenging staff’s negative stereotypes about young fathers.

**Discussion:**

Despite the small sample size, the results of this study nevertheless contribute to debates in the field regarding appropriate strategies for engaging young fathers by informing professional practice.

## Significance

*What is already known* Recruiting and retaining young fathers in programs for pregnant and parenting adolescents is difficult. Additional strategies informed by the lived experiences of young fathers and program staff are needed to improve engagement.

*What this study adds*Practitioners developing programs for young men should create explicitly father-focused recruitment messaging shared through participant word of mouth and case managers’ existing community ties. To retain young men, programs should incorporate strategies that provide emotional support for participants to navigate relationships with parents, co-parents, and friends; allow fathers to guide programming; and provide opportunities to connect with other young fathers.

## Introduction

Adolescent fathers are an understudied and underserved population (Weber 2012). Despite existing links between teen fatherhood, involvement in risky behaviors, and poor academic outcomes (Barlow et al. 2011; Bunting and McAuley 2004; Buston et al. 2012; Miller-Johnson et al. 2004), teen pregnancy and parenting support programs tend to be designed around the needs of teen mothers. Interventions designed for and with young men are rare, particularly in relation to teen rapid repeat pregnancy prevention and the promotion of positive parenting and sexual relationships.

Research shows that mainstream services generally do not effectively engage with fathers (Maxwell et al. 2012; Scourfield et al. 2014) and that fathers do not see the available services as relevant to them; the perception that “parenting” and family programs are designed for and often delivered by women is powerful (Katz et al. 2007). A study of young fathers’ experiences of the Family Nurse Partnership program (Ferguson 2016) found that non-engagement arose from a combination of service delivery issues as well as from complexities around their own vulnerabilities and prior negative service engagement experiences. Indeed, young fathers are frequently stigmatized as absentee and uninvolved in parenting (Duncan 2007), which results in young fathers commonly reporting real or perceived experiences of exclusion or bias by mainstream services (Fletcher and Visser 2008; Ross et al. 2012). This compounds with a prevalent belief that seeking or receiving help may be regarded as unmasculine (O’Brien et al. 2005) to produce negative attitudes toward engaging in programming.

Although these barriers represent a few of many potential reasons that efforts targeted at young fathers have reported struggling to engage participants, research as to what works to successfully engage this group is slim. Preliminary evidence suggests key factors driving success may include the degree to which young men can build a trusting relationship with the service providers and the extent to which they feel they are in control of the help they are receiving (Axford et al. 2012; Katz et al. 2007). Additional work in this area is needed because the available evidence suggests that young men’s levels of service engagement remain low (Davies 2016).

To support practitioners in overcoming the barriers described above and to further elucidate successful approaches, this manuscript presents the findings of a small qualitative study that aimed to gather lessons learned from one program’s staff and participants on effective strategies for engaging young fathers.

### Program Details

Five MI-APPP- local agencies were funded by the Michigan Department of Health and Human Services’ (MDHHS) Michigan Adolescent Pregnancy and Parenting Program (MI-APPP). They were charged with implementing the evidence-informed Adolescent Family Life Program-Positive Youth Development (AFLP-PYD) case management intervention and additional locally driven supplemental services with expectant and parenting young men and women. AFLP-PYD is an established voluntary case management approach for expectant and parenting young mothers and fathers that emphasizes building upon the participants’ strengths and resources, with the end goals of preventing future unplanned pregnancies and promoting positive youth and family outcomes. Each agency supported between one to three case managers and between one to three other staff (coordinators, outreach specialists, and support staff) with MI-APPP funding. Participants are generally recruited through word of mouth; referrals by school officials or other community-based organizations; and through other community outreach work (e.g., hosting public events, posting flyers, visiting spaces where youth gather).

Once fully briefed on the program and enrolled, participants are assigned to a case manager who helps them achieve their goals, usually related to education, parent and child health, adult connectedness, and overall family wellness. Case managers are expected to maintain a caseload of approximately 25 clients with whom they meet at least twice a month, with no time limit on clients’ enrollment. The enrolled expectant and parenting youth work with their case managers on specific exercises and tools intended to support youth in achieving their goals and achieving the other outcomes of the program. Supplemental services, conversely, vary greatly from site to site based on local participant needs and interests; they are intended to be a secondary focus to case management activities. Although supplemental services generally focus on similar topical areas, attendees may be AFLP-PYD participants; other pregnant and parenting youth; or other persons that provide support to this group (e.g., grandparents, friends, romantic partners, mentors). Some supplemental activities are gender specific, aiming to target specifically young mothers or young fathers.

Considering the scarcity of suitable services for expectant and parenting young men, the state prioritized the intentional inclusion of young fathers in programming, developing and delivering tailored, youth- and gender-responsive services to young fathers (in addition to young mothers) through the MI-APPP. Sites are supported in the often challenging task of engaging fathers through ongoing coaching and peer-to-peer learning opportunities for young father–serving case managers across the sites.

To ensure MI-APPP case management and supplemental services were well suited to the target populations, the program conducted a needs assessment during its initial planning year that specifically explored primary and secondary data on the needs and experiences of young fathers. This gender-separated data informed the development of state and local programming and recruitment/retention strategies that were intended to be responsive to fathers’ needs. Further refinements therein occurred over the first few years of the program, resulting in the adoption of a variety of innovative approaches intended to get young men interested and engaged in the program. At the time of this study, MI-APPP had served more than 150 young fathers and 800 young mothers, most of whom were ages 15–21, African American, and residents of urban areas. More than 75 of these young men had taken part in AFLP-PYD case management, with an average tenure in case management of one year (360 days). The other young men had participated solely in the aforementioned supplemental services.

The following study presents the results of a one-time small qualitative exploration intended to document recruitment and retention strategies used with young fathers and to identify those that were perceived to be most successful by MI-APPP participants and staff.

## Methods

MI-APPP’s MDHHS administrators, MI-APPP case managers, and young male participants were recruited for focus groups that took place in January 2018. One group was held with the creators and managers of MI-APPP at MDHHS namely, the program director, her unit manager, and their part-time case management consultant. This group was facilitated by MI-APPP’s evaluation team and focused on the origins and perceived success of the fatherhood engagement strategies developed and/or mandated by this state-level team.

The other two groups were designed to jointly engage young fathers and their case managers. The six MI-APPP case managers that had male participants on their caseload at this time were eligible to participate. These six individuals (from four MI-APPP sites) were invited to participate in the groups and to recruit their young father clients. Five case management staff elected to participate; the sixth case manager experienced travel challenges that precluded participation. The participating case managers recruited a convenience sample of 11 of the 13 young fathers who had an “Active” case management status at the time of the meeting, meaning they had been enrolled and had not yet exited case management. The young men in attendance had been engaged with MI-APPP for a range of 2 to 36 months, and the majority identified as African American. Although data on the *average* length of time engaged are not available for this specific sample, the average time between enrollment and exit in case management across sites was approximately 12 months (360 days). Each young father received a $25 VISA Gift Card incentive to compensate him for his time in the focus group.

Each of these two joint focus groups were composed of two or three case managers and the young men they had recruited from their caseloads. This mixed-groups approach was used upon the recommendation of a male engagement specialist who had observed that the trusting nature of the relationships between the case managers and their clients seemed to promote greater conversational participation from the young men than he had witnessed in groups solely composed of young men who are strangers. Each group was facilitated by men of color to cultivate a sense of trust through shared identity. These groups asked about case managers’ recruitment and retention strategies and about the deciding factors for fathers to engage and stay involved in the program.

All focus groups were audio recorded with the consent of all participants and were transcribed by a third-party transcriptionist. All procedures were approved by the MI-APPP evaluation team’s Institutional Review Board to ensure protection of human subjects.

### Analytic Procedures

The first and second authors (hereafter, authors) conducted a qualitative analysis of the focus group transcripts following the methods of Taylor and Bogdan (1998). From an initial read-through of transcripts and youth recruitment/retention literature, the authors developed a coding scheme based on emerging ideas and themes. Each focus group transcript was then coded independently using the agreed-upon coding scheme, line by line, using the qualitative software NVivo. Once the data from the focus groups were coded, authors reviewed the data to develop interpretations and conclusions. A summary of findings was shared with stakeholders as a form of member checking to provide participants an opportunity to verify, challenge, or confirm initial findings (Cohen and Crabtree 2006). The authors partnered with an MDHHS male engagement specialist in May 2018 to conduct the member checking process with 12 young fathers who were actively engaged in MI-APPP at that time. (Four of the 12 had been present at one of the focus groups.) The feedback received from youth who participated in member checking confirmed the authors’ initial findings.

After the findings were confirmed, the authors compared the themes to existing frameworks around recruitment and retention to understand the extent to which these recommendations had been made elsewhere in the literature. The authors determined the strategies were a natural extension of the high-level strategies described in the toolkit “Five Strategies for Successful Recruitment and Retention of Children and Families in Human Service Programs” (Barnes-Proby et al. 2017) to work with a specific group, namely, young fathers. This framework describes a set of evidence-based or promising strategies for client engagement developed from a synthesis of extant literature and consultation with national experts. It provides a good starting place to understand the components of a strong and comprehensive initial and ongoing recruitment and retention plan. Presenting key results of the present study in this framework demonstrates the connections between findings of the study regarding the engagement of young men and general best practices for recruitment and retention of human service clients. In this way, father-specific strategies are situated within the context of a more comprehensive engagement plan for practitioners. Each strategy in this framework is briefly summarized as it is presented in the results section, followed by the presentation of this study’s related findings.

## Results

### Strategy One: Outreach

The first strategy described in the framework is to *conduct outreach to raise awareness of the program* by developing positive community relationships and communicating the potential value of services.

#### Intentionally and Clearly Presenting that the Program Serves Young Fathers

Intentionally defining fathers as a target population was critical to the program’s success in engaging young men, according to all MDHHS participants.. Although the state-level and local messaging with adolescent-friendly messages for potential program participants was mostly designed appeal to both young mothers and young fathers, case management and young father participants discussed how neutral materials can be interpreted as targeting moms because of the assumption that “pregnant and parenting teens” are women. Therefore, it is also important to include elements to appeal to males, such as a male voice on radio ads or explicitly saying the program serves dads.

We serve dads, whereas [other programs] possibly serve them as “the other person in the room.” But [they] don’t collect any metrics, don’t do any fatherhood focused things, don’t really even sometimes address them as a critical piece to that developing child. MDHHS Staff Member

#### Ensuring Recruitment Messages Celebrate Involved Fatherhood

All three MDHHS participants described how the results of focus groups with young fathers conducted for the foundational MI-APPP needs assessment in 2013 revealed that young men *wanted* to be involved in their children’s lives, contrary to prevailing societal messages about absentee fathers. Three case managers from across the two joint focus groups described working hard to communicate to potential participants that they reject such negative stereotypes, instead highlighting fathers’ strengths and how their children can be a powerful motivator for them to better their personal and professional lives.

I think [the needs assessment results] changed the way that we kind of navigated once we got engaged with fathers. …We weren’t trying to encourage them; they’re already encouraged. It was like, “I know you want to be a good dad. I know you want to finish school. I know you want to be a provider” versus “You really need to provide for this child. You really need to finish school.” MDHHS Staff Member

### Strategy Two: Develop and Maintain Referral Sources

The second strategy in the framework is to *develop and maintain relationships with formal and informal referral sources* by building a strong collaboration with potential referral sources and to increasing the reach to families that might benefit.

#### Promoting Word of Mouth Referrals

Participants in all three focus groups reported that one of the more successful recruitment activities was promoting word of mouth among current program participants, including by incentivizing young women already enrolled in the program to bring their male partners and/or children’s fathers. One MDHHS staff perceived that when both parents of a child were involved with the program, they were both more likely to remain engaged over time, whether they were still romantically involved or not. This staff believed that this situation was often better for the entire family unit and ultimately represented a cost-effective recruitment and retention strategy for the program overall. All case managers in both joint focus groups also shared they had had some recruitment success through encouraging young men already enrolled in the program to invite their expectant and parenting male friends to participate.

#### Leveraging Social Capital

Leveraging case managers’ existing social capital was seen to be key to successfully spreading information about the program to school staff, local businesses, and other potential referral sources that may serve youth. Four case managers mentioned several examples wherein their connections within their community made the processes of conducting initial cross-agency outreach, receiving warm referrals, and directly recruiting youth they already knew much more successful. Two MDHHS participants mentioned believing this social capital approach to be most successful when the case managers possessed qualities such as professional experience working with young men, lived experience as a young parent, and shared identities with the population of interest.

### Strategy Three: Design Needs-Responsive Program Infrastructure

The third strategy in the framework is to *design program infrastructure and procedures that consider families’ needs* by providing services that are appropriate and accommodating to convey the value of participation and by addressing logistical, attitudinal, or other types of barriers to engagement.

#### Providing Trainings that Challenge Staff Bias

Challenging bias in initial and ongoing staff trainings delivered by state program staff who have expertise in working with hard-to-reach adolescent males was seen to promote male engagement. For example, two MDHHS participants described that the provision of extensive training and coaching to MI-APPP staff and other youth-serving providers around the state helped the professionals recognize how negative stereotypes about young fathers may manifest in their own work and to begin to overcome this bias.

#### Offering Opportunities for Young Fathers to Connect

Offering targeted opportunities for young fathers to connect with other fathers provided them support and a sense of belonging. Whether paired with parenting education components or for purely social engagement, this approach was reported to promote retention in all three focus groups.

[A fatherhood event] was inspiring to me because I never knew that many fathers that had went through a lot of stuff that I have went through myself. And then it was good for me because I was learning from them what not to do and what I can do to help me improve. Young Father

All the case managers and several young fathers in both joint focus groups described both peer-to-peer opportunities and multigenerational fatherhood-focused gatherings (involving participants’ family members or other older men) as significant for the young men because they normalized the experience of connecting around fatherhood. Additionally, both case managers and young fathers believed such gatherings may have promoted recruitment into case management because they provided a low-risk opportunity for other young men to try out the program in an environment where they felt comfortable among their peers before jumping into the more intensive one-on-one environment of case management.

### Strategy Four: Engaging and Supporting Families

The fourth strategy in the framework is to *engage and support families participating in the program* by establishing and maintaining positive rapport with families throughout program implementation to secure ongoing buy-in and engagement and by ensuring that families feel valued and connected to program staff.

#### Supporting Youth’s Goals

All case managers shared that supporting youth in taking concrete steps toward making and achieving goals appeared to help keep young men engaged, whether they were getting a job and managing their finances, finding housing, or improving their co-parenting relationship.

So, there is this stigma of “Just go get some money, and throw money to the mom, and you’ll be considered a good dad.” And we hold them to a higher expectation. “How much time did you spend with the child? …So, what type of schedule are you setting up where you can actually spend time with them?” Holding them to parenting and not just being this provider. MDHHS Staff Member

Multiple young fathers in both joint focus groups expressed that this component of the intervention helped them make progress toward becoming independent, healthy parents and that seeing the value of this improvement was one of the reasons they stayed engaged with the program.

#### Providing Emotional Support

Providing nonjudgmental emotional support kept youth participants in both joint focus groups engaged by helping the young men navigate their relationships with their children, their children’s other parent(s), significant others, and their own parents.

So, when it comes to situations like real deal, serious situations with your girl and you don’t know who to talk to. You don’t want to talk to [your] moms, you don’t want to put her in your business, that’s why you turn to [case manager] because she ain’t going to judge you. She is good with the advice. Young Father

Young fathers in both groups also described their relationships with their case managers as the most important benefit of the program and a core reason that they remained involved.

### Strategy Five: Monitor Enrollment, Retention, and Services

The fifth strategy in the toolkit framework is to *continuously monitor family enrollment and retention and quality of services* by determining which families are enrolling, maintaining, and exiting the program and why and modifying program processes accordingly.

#### Minimum Caseloads

The requirement that each program site have a minimum percentage of young men on its caseload was set to guide program priorities around male engagement. All three MDHHS Staff Members and one case manager reported that quarterly reporting on caseloads, broken down by gender and type of engagement (case management versus supplemental services), seemed to help ensure local sites are meeting their young father enrollment numbers. Sites are supported in meeting this requirement via ongoing coaching and technical assistance from a male engagement expert and regular in-person or virtual peer-to-peer learning opportunities for young father–serving case managers across the MI-APPP sites. Three case managers and two MDHHS staff members shared that these peer-to-peer opportunities help to create a space for the case managers to share lessons learned and brainstorm joint-site approaches.

#### Centralizing Young Fathers’ Feedback

Cultivating a culture that centralizes young fathers’ feedback means consistently asking male participants what they want and authentically attempting to meet these needs in group and individual activities. Two MDHHS participants, two case managers in one joint focus group, and two young fathers in the other joint focus group described how this information gathering has occurred through formal methods (e.g., focus groups, panels at conferences) and informal methods (case managers asking fathers what they want) throughout the life of the program. These case managers shared that monitoring fathers’ experiences in this way led certain sites to make changes, such as focusing on providing fun outings (e.g., sporting events) as incentives for participation and creating fathers-only spaces where young men can discuss their shared experiences.

## Discussion

This paper has focused on lessons learned from an intervention that successfully recruited and retained traditionally hard-to-reach young fathers in an intensive case management program. Although preliminary in nature, this study’s findings represent the voices of critical stakeholders in the process of recruiting young fathers—the state agency that creates and implements the policies, the local staff who reach out and engage participants, and the young men themselves. Its results contribute important information to the very sparse literature on this underserved group and to practitioners looking for transformative strategies for engaging young fathers. Furthermore, presenting the results within the Barnes-Proby et al. (2017) framework for client engagement situates these findings within the broader discussion of recruitment and retention of hard-to-reach populations.

Practitioners developing programs for young men should consider more deeply how the needs of young fathers may differ from those of young mothers and older fathers and create responsive strategies. For example, outreach messages that explicitly said the program was intended for dads were critical to engaging fathers, a finding that echoes previous work (Buston 2018). Staff participants also identified regular participant feedback processes as essential to tailoring the program offerings to the needs of the young men. These results suggest that assessment and response to fathers’ needs should occur in an ongoing fashion throughout the life of the program and involve direct input from the target participants themselves as much as possible. This aligns with previous research suggesting that successful engagement may hinge on participants’ sense of control in the helping relationship (Axford et al. 2012; Katz et al. 2007).

Actively dispelling myths around father disengagement with youth and working against providers’ own bias against young fathers were other important components of the success of the program. Case managers instead intentionally cultivated and communicated a belief that the young men wanted to be caring and engaged and that their children can be part of what motivates them to achieve personal and professional success. This further supports new work that similarly highlighted young men’s aspirations to be “good fathers” who want to be present for their child(ren) in ways their own fathers had often not been able to be (Buston 2018). Providers should incorporate these efforts in their own work, demonstrating to participants how attending their services could be helpful in this pursuit.

The fathers in this study identified the relationships they had built with their case managers as one of the most important contributors to continued participation, concurring with existing literature (Axford et al. 2012; Katz et al. 2007; Pfitzner et al. 2017). They also identified supplemental service opportunities that provided spaces to connect with and learn from other fathers as a much-appreciated recruitment and retention driver. Indeed, these less emotionally risky, low-pressure group activities were seen by young men and case managers alike as a space where newly recruited young fathers could test out the culture of the program before committing to intensive case management. This suggests such opportunities for connection may be a promising strategy for engaging young men who may be questioning the fit of the program. These findings also suggest that young men are craving meaningful connection with adults and peers. Future research efforts should dive more deeply into promoting this connection because it could present an important key to recruiting young fathers and keeping them engaged over time.

Although this study represents critical perspectives in fatherhood engagement work, there are limitations worth noting. By focusing on identifying successful strategies, the study was not able to produce results contributing to understanding challenges or unsuccessful strategies with this population. Additionally, the study’s small sample size and focus on a single program limit its generalizability to the broader field. Further, although the youth sample in this study contains a considerable proportion of fathers in the program at the time of the study, the sample may not be representative of the full sample of MI-APPP participants or the wider population of young fathers. Finally, the structure of the joint case manager and young father focus groups was not designed to elicit information on the prevalence of any given perspective or to distinctly differentiate between the perspectives of these two groups. Future explorations of effective recruitment and retention techniques for case management programs should include a larger number of both fathers and staff members to allow for greater reassurance of saturation of themes, consider an alternative focus group structure to differentiate between these perspectives (that still prioritizes authentic and engaged participation), and include multiple similarly intensive interventions to allow for engagement lessons learned across programs.
